# Bioenergetic and Autophagic Characterization of Skin Fibroblasts from *C9orf72* Patients

**DOI:** 10.3390/antiox11061129

**Published:** 2022-06-08

**Authors:** Maria Isabel Alvarez-Mora, Gloria Garrabou, Tamara Barcos, Francisco Garcia-Garcia, Ruben Grillo-Risco, Emma Peruga, Laura Gort, Sergi Borrego-Écija, Raquel Sanchez-Valle, Judith Canto-Santos, Paula Navarro-Navarro, Laia Rodriguez-Revenga

**Affiliations:** 1 Biochemistry and Molecular Genetics Department, Hospital Clinic of Barcelona, 08036 Barcelona, Spain; mialvarez@clinic.cat (M.I.A.-M.); barcos@clinic.cat (T.B.); eperuga@clinic.cat (E.P.); lgort@clinic.cat (L.G.); pnnavarro253@gmail.com (P.N.-N.); 2 CIBER of Rare Diseases (CIBERER), Instituto de Salud Carlos III, 08036 Barcelona, Spain; garrabou@clinic.cat (G.G.); jcanto@clinic.cat (J.C.-S.); 3 Institut d’Investigacions Biomèdiques August Pi i Sunyer (IDIBAPS), 08036 Barcelona, Spain; rsanchez@clinic.cat; 4 Muscle Research and Mitochondrial Function Laboratory Cellex-IDIBAPS, Faculty of Medicine and Health Sciences, University of Barcelona, Internal Medicine Department, Hospital Clinic of Barcelona, 08036 Barcelona, Spain; 5 Bioinformatics and Biostatistics Unit, Principe Felipe Research Center (CIPF), 46012 Valencia, Spain; fgarcia@cipf.es (F.G.-G.); rgrillo@cipf.es (R.G.-R.); 6 Alzheimer’s Disease and Other Cognitive Disorders, Neurology Service, Hospital Clinic de Barcelona, 08036 Barcelona, Spain; borrego@clinic.cat

**Keywords:** C9orf72, autophagy, SUMOlyation, bioenergetics

## Abstract

The objective of this study is to describe the alterations occurring during the neurodegenerative process in skin fibroblast cultures from *C9orf72* patients. We characterized the oxidative stress, autophagy flux, small ubiquitin-related protein SUMO2/3 levels as well as the mitochondrial function in skin fibroblast cultures from *C9orf72* patients. All metabolic and bioenergetic findings were further correlated with gene expression data obtained from RNA sequencing analysis. Fibroblasts from *C9orf72* patients showed a 30% reduced expression of C9orf72, ~3-fold increased levels of oxidative stress and impaired mitochondrial function obtained by measuring the enzymatic activities of mitochondrial respiratory chain complexes, specifically of complex III activity. Furthermore, the results also reveal that *C9orf72* patients showed an accumulation of p62 protein levels, suggesting the alteration of the autophagy process, and significantly higher protein levels of SUMO2/3 (*p* = 0.03). Our results provide new data reinforcing that *C9orf72* cells suffer from elevated oxidative damage to biomolecules and organelles and from increased protein loads, leading to insufficient autophagy and an increase in SUMOylation processes.

## 1. Introduction

An intronic hexanucleotide repeat expansion (G_4_C_2_) in the *C9orf72* gene is the most common genetic cause of amyotrophic lateral sclerosis (ALS) and frontotemporal dementia (FTD) (referred to as C9ALS/FTD) [1,2]. While unaffected individuals carry less than 10 G_4_C_2_ repeats, C9ALS/FTD patients carry anywhere between 30 and 1600 repeats [1,2]. The pathogenic mechanism may involve the loss of C9orf72 protein function, a toxic RNA gain-of-function mechanism and gain of toxic function from dipeptide repeat protein (DPR) generated by repeat-associated non-ATG (RAN) translation [3]. RAN translation occurs in all three reading frames both in the sense and antisense strands, leading to the translation of five dipeptide repeats (DPRs) [4]. Both gain-of-function models are supported by observations of the pathologic aggregation of intranuclear RNA foci and cytoplasmic inclusions of the DPRs in patient neurons [5,6].

Neuropathologically, C9ALS/FTD cases exhibit abnormal neuronal and oligodendroglial inclusions of the transactive response DNA-binding protein 43 KDa (TDP-43) [7,8]. Subjects with *C9orf72* expansion also present a neuropathological hallmark consisting of the presence of small ubiquitin/p62-positive and TDP-43-negative cytoplasmic inclusions in the cerebellum, hippocampus and neocortex, which contain DPR proteins [7].

One of the main reasons implicated in the accumulation of protein aggregates may be defective autophagy [9]. There are three major sub-types of autophagy: microautophagy, chaperone-mediated autophagy and macroautophagy [10]. Macroautophagy (hereafter referred to as autophagy) represents the major degradative autophagy pathway capable of degrading both protein aggregates and defective organelles [10]. Macroautophagy is a dynamic process that comprises multiple steps. Briefly, polyubiquitination is the signal for the recruitment of adaptor proteins, such as p62, which allows the binding of the microtubule-associated protein 1 light chain 3 (LC3BII) in the forming autophagosome to initiate the sequestration of intracellular cargos and the subsequent clearance upon fusion with the lysosome [11]. Apart from autophagy, other pathways, such as SUMOylation (an important post-translational modification), are implicated in protein assembly and disassembling [12]. Small ubiquitin-related modifier (SUMO) proteins function as reversible protein modifiers. Growing evidence documents a link between protein SUMOylation and critical process, including cellular localization, chromatin organization, genome stability, signal transduction, protein–protein or protein–DNA interactions, and transcriptional regulation [13]. The relationship between SUMOylation and neurodegenerative disorders has been well-studied [12], and SUMO proteins have been described as major inclusion proteins [14] or enriched in post-mortem brain samples of patients affected by neurodegenerative disorders [15,16]. In relation to C9ALS/FTD, in the last few years, some studies have reported a relationship between TDP-43 pathology and SUMOlyation [17], depicting the importance that this pathway might have in the pathogenesis of the disease.

In an attempt to provide further insights into the alterations occurring during the neurodegenerative process in C9ALS/FTD skin fibroblasts, we characterize the oxidative stress, autophagy flux, SUMO2/3 protein levels as well as the mitochondrial function in skin fibroblast cultures from *C9orf72* patients. All metabolic and bioenergetic findings are further correlated with gene expression data obtained from RNA sequencing analysis.

## 2. Methods

### 2.1. Subjects

Patients with C9ALS/FTD (3 females and 1 male) and control subjects with normal G_4_C_2_ repeat expansion (<30 G_4_C_2_) (2 females and 2 males) were recruited for this study. All patients with C9ALS/FTD were clinically evaluated at the Alzheimer’s disease and other cognitive disorders unit of the Neurology Service of the Hospital Clinic Barcelona, Spain, and encompass patients who fulfilled criteria for ALS or FTD. The control cohort consisted of healthy age-matched individuals with no clinical evidence of main neurodegenerative diseases that voluntarily underwent skin biopsy. All samples included were of Caucasian origin. Table 1 summarizes the clinical and molecular findings of the subjects recruited in the study. No significant difference was observed in sex or age composition between both groups (*p* *=* 0.465 and *p* = 0.883, respectively).

All participants provided written informed consent for testing and the use of their phenotypic/clinic and genetic data. The study was approved by the Ethics Committee of the Hospital Clinic of Barcelona, following the guidelines of Helsinki declaration (2013).

### 2.2. Fibroblasts Cell Culture

Skin biopsies were obtained using a 3 mm punch from 4 *C9orf72* patients and 4 control individuals recruited from the Dermatology Department of the Hospital Clinic of Barcelona. Under sterile conditions, the biopsy was cut and plated at 37 °C with a 5% CO_2_ atmosphere in T25 flasks in minimum essential media (MEM) 13% containing 500 mL MEM (Gibco, ThermoFisher Scientific, Madrid, Spain) and 75 mL newborn calf serum (Gibco, ThermoFisher, Madrid, Spain) supplemented with 0.30 mL penicillin (Gibco, ThermoFisher Scientific, Madrid, Spain) and 0.30 streptomycin (Gibco, ThermoFisher Scientific, Madrid, Spain). A trypan blue exclusion test was used to quantify the number cell viability and cell counts using a hemocytometer. No differences in cellular viability were detected when comparing *C9orf72* and control cell cultures. All functional assays were performed in cells between passage 5 and 10.

### 2.3. C9ORF72 and SUMO2/3 Protein Quantification

Cell pellets obtained from cultured fibroblasts were homogenized with RIPA buffer (SDS 0.1%, NP40 1% and sodium deoxycholate 0.5% in PBS) containing protease inhibitors (cOmplete™, Mini, EDTA-free Protease Inhibitor Cocktail 4693159001, Roche, Indianapolis, IN, USA). Cell lysates were subjected to SDS-PAGE and electroblotted. Proteins were visualized by immunostaining with antibodies against C9orf72 1:5000 (Abcam, Marid, Spain, ab221137) SUMO2/3 1:1000 (Abcam, Marid, Spain, ab3742) and β-actin 1:5000 (A5441, Merck KGaA, Darmstadt, Alemania). Colorimetric detection (1708235 Opti-4CNTM Substrate Kit, Bio-Rad, Hercules, CA, USA) and ImageJ software were used for the densitometry analysis of protein expression.

### 2.4. Oxidative Stress Assessment

Lipid and protein peroxidation levels are indicative of the reactive oxygen species (ROS)-derived oxidative damage in cell lipid and protein compounds. Lipid peroxidation levels were quantified using the BIOXYTECH^®^ LPO-586™ colorimetric assay (Oxys International Inc., Portland, CA, USA). The levels of peroxides derived from fatty acid oxidation (malondialdehyde (MDA) and 4-hydroxyalkenal (HAE) were analyzed by spectrophotometry. The results were subsequently normalized by protein content and expressed as μM MDA+ HAE/mg protein.

Carbonyl groups, the protein oxidation products, were measured by Western blot analysis using the commercially available OxyBlot kit assay, according to the manufacturer’s specifications (Merck KGaA, Darmstadt, Alemania). The manufacturer’s instructions were followed and 20 μg of protein was derivatized for each sample. Results were quantified by densitometry (Image Quant TL Software, GE Healthcare, Barcelona, Spain) and normalized by β-actin content, as a cell protein loading and house-keeping marker.

### 2.5. Mitochondrial Metabolism Assay

In order to measure mitochondrial activity, mitochondrial respiratory chain enzymatic activities (MRC) were quantified. The enzymatic activities of mitochondrial complexes I, II, III, IV, I+III and II+III were spectrophotometrically measured at 37 °C in fibroblasts, as reported elsewhere [18]. Citrate synthase (CS) activity was also spectrophotometrically determined at 37 °C in fibroblasts, as it is considered a reliable marker of mitochondrial content [19]. All enzymatic assays were performed following the national standardized methods and were run in parallel with internal quality controls [20]. Changes in absorbance were recorded in a Uvikon XS spectrophotometer (Secomam-Aqualabo Group, Alès, France) through LabPower v4 software. Results were expressed as nanomoles of consumed substrate or generated product per minute and milligram of protein (nmol/minute·mg protein). All enzymatic activities were normalized by CS activity.

### 2.6. Autophagic Flux Analysis

Bafilomycin A1 (Merck KGaA, Darmstadt, Alemania) reagent was added to the medium at a final concentration of 0.1 µM, and cells were incubated for 4 and 8 h at 37 °C in the presence of 5% CO_2_. Bafilomycin A1 is a proton pump inhibitor that neutralizes lysosomal pH preventing the fusion of autophagosomes with lysosomes and, thus, allows the monitoring of autophagosome synthesis [21]. Briefly, fibroblasts were lysed with RIPA buffer (Merck KGaA, Darmstadt, Alemania) plus protease inhibitor cocktail (ThermoFisher Scientific, Madrid, Spain) followed by shaking and centrifugation at 16,100× *g* at 4 °C for 10 min. Soluble fractions were kept at −80 °C until Western blot analysis. Electrophoresis and blotting were performed, as reported elsewhere [18]. Blots were probed with antiSQSTM1/p62 (ab155686, Abcam) and anti-LC3B (#2775, Cell Signaling Technology, Leiden, The Netherlands) antibodies. LC3BII is considered a marker of autophagosome number and p62 is a ubiquitin-binding scaffold protein that labels molecules and organelles that need to be degraded. The p62 protein acts as a cargo receptor that is recruited to autophagosomes through LC3BII interaction [22]. According to the manufacturer’s protocol, the SYPRO Ruby Protein Blot Stain (ThermoFisher Scientific, Madrid, Spain) was used to obtain the total protein content. Mitochondrial content was estimated by Western blot through the measurement of voltage-dependent anion channel (VDAC) protein content relative to the β-actin signal, as previously described [23]. VDAC protein is the most abundant protein on the outer membrane of mitochondria, one of the key proteins that regulate mitochondrial function, and a widely accepted marker of mitochondrial amount. The intensity of signals was quantified by densitometric analysis (Image Quant TL Software, GE Healthcare). Results were expressed as p62 and LC3BII protein levels normalized by the total cell protein content (using SYPRO staining) or by mitochondrial content (using VDAC content).

### 2.7. Functional Enrichment Analysis

RNA sequencing data from the recruited sample set was available from previous studies (unpublished data). We carried out a functional enrichment analysis on the differential expression profile between the C9ALS/FTD and control samples. We applied the Gene Set Analysis (GSA) method implemented in the mdgsa R package with the Reactome pathways as functional annotations [24]. The *p*-values were corrected with the Benjamini and Hochberg method [25], considering significant those pathways with an adjusted *p*-value lower than 0.05. The GSA method uses a logistic regression model, so significant and positive log odds ratios (LOR) indicate upregulated pathways, while significant and negative LOR indicate downregulated pathways.

### 2.8. Accession of Data

All data underlying the findings described in the manuscript are fully available without restriction and upon request.

### 2.9. Statistical Analysis

Results are expressed as means ± the standard error of the mean (SEM). Statistical analysis was performed using the non-parametric Mann–Whitney U, x-square test and Fisher’s *t*-test when required, using commercially available software (SPSS-PC, version 19; SPPSS Inc., Chicago, IL, USA). Significance was accepted for asymptotic bilateral *p*-values below 0.05.

## 3. Results

### 3.1. Fibroblasts from C9ALS/FTD Patients Show Reduced C9orf72 Protein Levels

**Figure 1:**
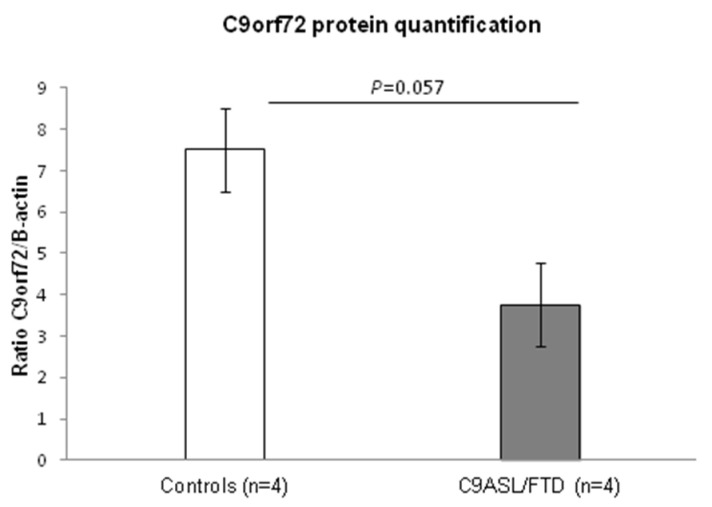
Analysis of C9orf72 protein levels in C9ALS/FTD patients (n = 4) and the control samples (n = 4) showed a 30% reduction in protein content in the patients. Results are expressed as means ± standard error of the mean (SEM).

A number of studies have described a reduction in C9orf72 transcript expression as a consequence of the hexanucleotide expansion, e.g., [1,26]. In order to characterize C9orf72 expression in total protein skin fibroblast lysates from patients and controls, an immunoblot analysis was carried out. An approximately 30% reduced expression of C9orf72 was detected in C9ALS/FTD patients compared to the control individuals (*p* = 0.057) (Figure 1).

### 3.2. Fibroblasts from C9ALS/FTD Patients Evidence Increased Levels of Oxidative Stress

**Figure 2:**
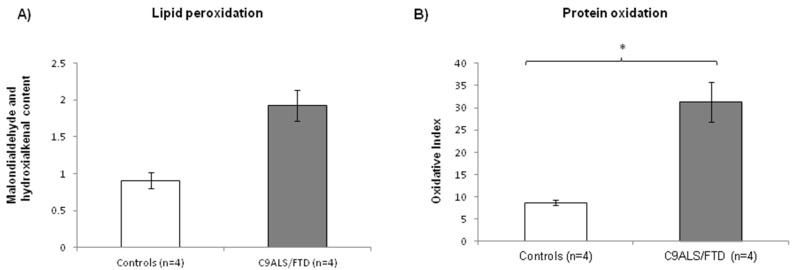
Oxidative stress measured through (**A**) lipid and (**B**) protein peroxidation in C9ALS/FTD patients and the control samples. C9ALS/FTD patients exhibited an upward trend in lipid peroxidation compared to the control fibroblasts and a significantly increase in protein oxidation levels (oxidative index as protein carbonyls). The results are expressed as means ± standard error of the mean (SEM). * *p* < 0.05.

Under conditions of oxidative stress, reactive oxygen species (ROS) production grows rapidly, leading to alteration of membrane lipids, protein and nucleic acids. Lipid peroxidation is the oxidative degradation of lipids. ROS are the major initiators of lipid peroxidation, whose byproducts cause direct damage to cell membranes. On the other hand, protein oxidation measured by carbonyl group formation on protein side chains is also a biochemical marker of cellular oxidative stress. In this paper, lipid and protein oxidation was measured as an indicator of ROS-derived oxidative damage. C9ALS/FTD patients exhibited an increase in both lipid and protein peroxidation compared to the control fibroblasts (Figure 2), presenting a 2-fold increase in lipid peroxidation (mean C9ALS/FTD = 1.92; mean control = 0.9; ratio = 2.12; *p*-value = 0.057) and a 3.5-fold increase in protein oxidation (mean C9ALS/FTD = 31.38; mean control = 8.7; ratio = 3.6; *p*-value = 0.016).

### 3.3. Fibroblasts from C9ALS/FTD Patients Evidence Mitochondrial Dysfunction and Altered Metabolism

To assess mitochondrial metabolism in fibroblasts from C9ALS/FTD patients, MRC complexes were analyzed. The enzymatic activities of the MRC complexes were analyzed, and the results evidence significantly decreased complex III activity in the skin fibroblasts from C9ALS/FTD patients compared to the controls (Table 2). These results might suggest that, under pathological conditions, an important mitochondrial dysfunction occurs, leading to higher oxidative stress damage and defective mitochondria accumulation.

Fibroblasts from C9ALS/FTD patients show an accumulation of p62 protein levels, suggesting the alteration of the autophagy process.

**Figure 3:**
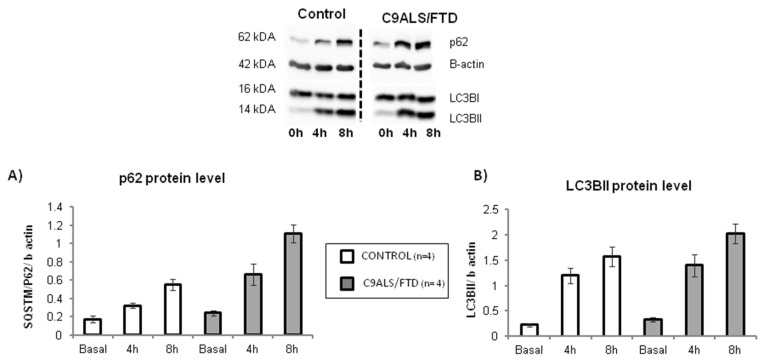
Autophagic flux in C9ALS/FTD patients and the control fibroblasts. (**A**) p62 and (**B**) LC3BII protein level at basal conditions (0 h) and under bafilomycin A1 treatment (4 or 8 h). The results are expressed as means ± standard error of the mean (SEM). No statistically significant results were obtained, although p62 protein levels were 2-fold increase in C9ALS/FTD patients compared to the control fibroblasts.

To determine the potential impact of C9orf72 expansion on autophagy, we quantified p62 and LC3BII protein levels, considered the starting and ending key-players of the autophagic process, respectively. No statistically significant differences were observed in C9orf72 fibroblasts compared to the controls (Figure 3) (*p* = 0.2 for p62 protein level at basal conditions or 0 h; *p* = 0.7 for LC3BII protein levels at basal conditions). In order to evaluate the autophagic flux, both proteins were further measured after bafilomycin A1 cell treatment. Bafilomycin A1 disrupts the autophagic flux by impairing the autophagosome–lysosome fusion and, thus, blocks autophagosome degradation. Therefore, this treatment allows the measurement of autophagosome synthesis, whereas both synthesis and degradation occur in the untreated samples. Two different times of bafilomycin A1 treatment (4 h and 8 h) were established. As shown in Figure 3A, p62 levels were increased after treatment in both groups (patients and control), evidencing the effect that this drug has in the autophagic flux. Although p62 levels at 4 and 8 h of treatment were higher in C9ALS/FTD compared to the control fibroblasts, no statistically significant differences were obtained (*p* > 0.05) (Figure 3A). Similarly, the analysis of LC3BII levels evidenced the same gradual increment for the controls and C9ALS/FTD patients with no significant differences (*p* > 0.05) (Figure 3B). These results suggest that, despite higher autophagic initiation (p62 labeling), autophagic flux is not finally increased in terms of autophagosome closure (LC3B-II staining) in skin fibroblasts from *C9orf72* patients, and thus, that cells accumulate autophagic cargo, such as aggressive aggregates or damaged organelles.

**Figure 4:**
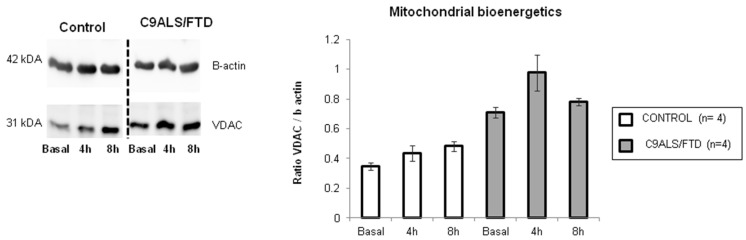
Mitochondrial bioenergetics in C9ALS/FTD patients and the control samples. VDAC protein content was quantified by Western blot and represented as a ratio of β-actin content. The results are expressed as means ± standard error of the mean (SEM). Although not statistically significant, fibroblasts from C9ALS/FTD patients showed a 2-fold increase in VDAC content at basal conditions and after bafilomycin treatment (4 h and 8 h).

In order to assess if the content of this autophagic cargo is damaged mitochondrial organelles, the VDAC content was measured in these samples. The comparison of all time-point analysis between the control and C9ALS/FTD groups (0, 4 and 8 h of BA1 treatment) indicated a non-significant trend toward increased levels of VDAC protein (Figure 4), suggesting an increased accumulation of damaged mitochondria in C9ALS/FTD patients.

### 3.4. Fibroblasts from C9ALS/FTD Patients Show Increased SUMO2/3 Protein Quantification

**Figure 5:**
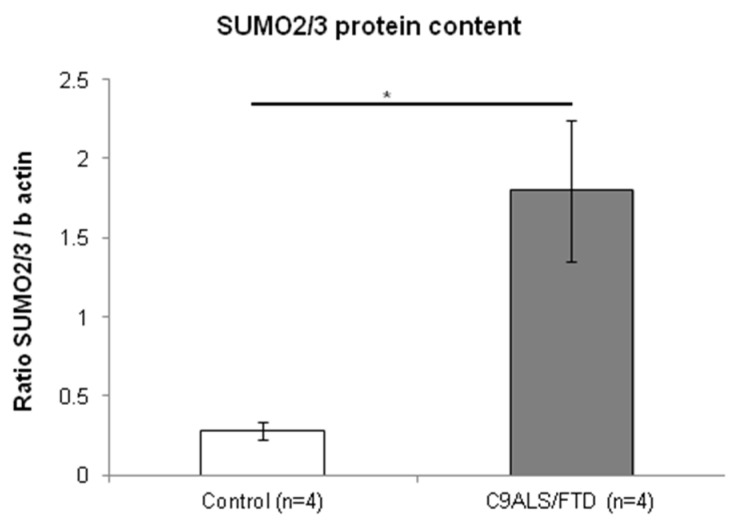
Fibroblasts from C9ALS/FTD patients show increased SUMO2/3 protein content. The results are expressed as means ± standard error of the mean (SEM). Significant *p*-values were considered for *p* < 0.05 (*).

SUMOylation has been described in close relation with autophagy and accumulating data indicate that interactions between SUMOylation and autophagy are important for maintaining cellular homeostasis [27]. In accordance with this observation, we aimed to assess SUMO2/3 protein levels in lysates from skin fibroblasts. The results reveal that C9ALS/FTD patients showed significantly higher protein levels of SUMO2/3 than the control group (*p* = 0.03) (Figure 5). Together, these results support the notion that C9ALS/FTD skin fibroblasts are subjected to high levels of cellular stress and that proteins are being accumulated.

### 3.5. Autophagy- and SUMOylation-Related Pathways Are Significantly Altered in Fibroblasts from C9ALS/FTD Patients

By using Reactome as a pathway database, we then investigated autophagy and SUMOylation pathways in expression profiles obtained from RNA sequencing data of fibroblasts from C9ALS/FTD patients. The functional enrichment analysis indicated that both processes are significantly dysregulated in the C9ALS/FTD transcriptome (Table 3). However, while those pathways involved in autophagy (i.e., macroautophagy or selective autophagy) showed a negative LOR, those in which SUMOylation is involved showed a positive one. Overall, these results evidence that autophagy-related pathways in fibroblasts from C9ALS/FTD patients are downregulated, whereas SUMOylation is upregulated.

## 4. Discussion

Protein aggregates and damaged mitochondria represent key pathological hallmarks shared by most neurodegenerative diseases. These protein aggregates are mainly targeted towards the autophagy–lysosome degradation pathway. Concurrently, it is thought that impaired autophagy can lead to the development of neurodegenerative diseases [28]. In fact, genetic mutations in core autophagy-related genes have been reported to be related to neurodegenerative diseases, such as frontotemporal dementia, Parkinson’s disease and Huntington’s disease [28].

In humans, *C9orf72* transcripts are detectable in most tissues, albeit most notably in all brain regions and the spinal cord [1,2]. In this regard, skin fibroblasts might be a suitable tissue model to assess functional cellular and bioenergetic impairment in C9ALS/FTD patients. In this context, previous studies have successfully used this tissue to mirror the alterations observed in C9orf72 neurons [29].

In our study, we used skin fibroblast cultures from C9ALS/FTD patients to characterize its pathological and functional properties. Since decreased protein levels of C9orf72 have been described in central nervous system areas and in some peripheral tissues of C9ALS/FTD patients [1,2,30], and C9orf72 haploinsufficiency has been described as a molecular mechanism responsible for the disease, we firstly quantified C9orf72 protein levels in our samples. Similar to what has been previously described, our results show a 30% C9orf72 reduction in C9ALS/FTD patients compared to the controls. Although it can be argued that these results were not statistically significant (*p* = 0.057), it has to be taken into account that it might be due to a small sample size effect, since our cohort consisted of four patients and four control samples. Recently, a study by Leskelä et al. [31] did not indicate decreased C9orf72 levels at either mRNA or protein level in the fibroblasts of *C9orf72* patients. Nevertheless, the approximation taken by these authors was different since they measured C9orf72 mRNA by quantitative PCR and protein levels in cells treated with lactacystin, a proteasome inhibitor.

Secondly, we aimed to assess oxidative stress in C9ALS/FTD cells by measuring lipid peroxidation and protein oxidation/nitration. Lipids, proteins, as well as DNA are examples of molecules that can be modified by excessive ROS in vivo. These parameters are considered as significant biomarkers reflecting the local degree of oxidative stress [32]. Our results evidence higher levels of oxidative stress in C9ALS/FTD skin fibroblast cultures compared to the controls, as reflected by the higher levels of both lipid peroxidation and protein oxidation. Although the results were only significant for protein oxidation (*p* = 0.02), both parameters showed a ~3-fold increase in samples from C9ALS/FTD patients compared to the controls. Together, these results might indicate hazardous stress burdens for proper cell function. In line with this, we found impaired mitochondrial function by measuring the enzymatic activities of the MRC complexes, specifically of complex III activity in C9ALS/FTD skin fibroblasts (Table 2).

A growing amount of evidence argues for oxidative stress as an important intracellular mediator of autophagy [33]. Autophagy contributes to clearing the cells of all irreversibly oxidized biomolecules. Moreover, several evidence demonstrates that the depletion of C9orf72 dysregulated autophagy [34,35,36] and suggests that the protein has a role in cellular trafficking and protein degradation [36,37,38]. Having shown that skin fibroblasts expressed lower C9orf72 protein levels and that they show significant levels of oxidative stress, we further characterized the autophagy flux by measuring p62 and LC3BII protein levels in a time course experiment treating cells with bafilomycin A1 (an inhibitor that prevents the fusion of autophagosomes with lysosomes and disrupts autophagic flux). The results show the slight accumulation of p62 in C9ALS/FTD patients compared to the controls under basal culture conditions and an increase in the treatment with bafilomycin A1. On the other hand, LC3BII levels were not changed, suggesting that fibroblasts from *C9orf72* patients initiate (by p62 labelling), but do not finish (by autophagosome closure through LC3B-II dilapidation), and trend to accumulate autophagosome structures. According to previously published research [39], these results support the idea that patients’ cells accumulate autophagic cargo that might not be properly recycled or cleared. Accordingly, autophagy-related pathways were found to be downregulated in expression profiles obtained from RNA sequencing data (Table 3). To check if that autophagic cargo is damaged mitochondria with dysfunctional MRC generating increased levels of oxidative stress, we measured VDAC content and found a 2-fold VDAC content increase after bafilomycin treatment. Although not statistically significant (*p* > 0.05), this result suggests the accumulation of deficient mitochondria in the autophagosomes of *C9orf72* patients.

The relationship between C9orf72 and autophagy has been widely studied. First, the C9orf72 protein has been involved in endocytic and phagocytic trafficking [34,40]. Second, it has been described as a member of a protein complex controlling not only early stages of autophagy, but also late stages, such as the autophagosome–lysosome fusion [35,36,37,41,42]. Finally, C9orf72 has also been described as colocalizing with stress granules, suggesting that it might also be relevant in selective protein aggregation macroautophagy [35,39]. Despite all these associations of C9orf72 with key autophagy players, the net impact on autophagy is still unclear. What seems clear, and our results reinforce it, is that cells from C9ALS/FTD patients show higher evidence of cellular stress and dysfunction that might affect its viability. Whether this dysfunction is a consequence of a decreased C9orf72 protein level or gain-of-function mechanisms or even due to other mechanistic effects remains still unclear. Recently, Biogen-Ionis announced that BIIB078, an investigational antisense oligonucleotide for C9orf72-associated ALS, did not show clinical benefit and patients at higher doses even worsened. This clinical result was observed even if the drug showed target engagement (reduction in polyGP) (https://investors.biogen.com/news-releases/news-release-details/biogen-and-ionis-announce-topline-phase-1-study-results, accessed on 10 March 2022). Even if there are no published peer-reviewed data available, this announcement suggests that the loss of function hypothesis should be reconsidered as the main hypothesis in the initiation of neurodegeneration, which is in line with our results. Nevertheless, another plausible scenario might be a synergistic mechanism since autophagy is needed to remove toxic gain-of-function products, such as DPRs [43,44].

Finally, our results also evidence significantly increased SUMO2/3 protein levels (*p* = 0.03), whose gain might reflect an accumulation of proteins destined to removal in skin fibroblasts for C9ALS/FTD patients. p62 and SUMO2/3 have been described as major components in neuronal inclusions detected in other neurodegenerative diseases, such as FXTAS or SCA7 [15,16]. Likewise to p62, SUMO2/3 are proteins implicated in the ubiquitin–proteasome system and their levels are stimulated in a stress-dependent manner [45]. RNA sequencing in fibroblasts from C9ALS/FTD patients also revealed the dysregulation of SUMOylation pathways. Of note, the SUMOylation and the SUMO E3 ligases SUMOylate target proteins pathway were found to be upregulated, which seems to be in agreement with the increase in SUMO conjugation activity observed in response to various stimuli, such as high levels of oxidative stress [46]. This study has two limitations. First, the small sample size, which might explain the lack of significance in some of the analyses, but also might increase the margin of error. The second limitation is related to the variability that autophagy and other metabolic pathways might have among different individuals, including healthy controls [47]. Despite these limitations, the fact that we detected statistically significant differences between groups, together with the previously described evidence, reinforce the veracity of the reported results in this paper.

## 5. Conclusions

In conclusion and on the basis of our results, it can be hypothesized that C9ALS/FTD cells suffer from elevated oxidative damage to biomolecules and organelles (e.g., mitochondria) and from increased protein loads, leading to an insufficient autophagy and increase in SUMOylation processes. Although the sample size is small and the observed difference could reflect a natural variation between the individuals, several evidences might reinforce these results, for instance, Sieller and co-workers [35] reported suboptimal autophagy with the notable accumulation of unresolved aggregates of the autophagy receptor P62/SQSTM1 in neuronal cultures. However, on the other hand, it could also be argued that the levels of oxidative damage observed as well as the amount of “defective” mitochondria can be well tolerated. Skin fibroblasts are cells that divide, and it might be possible that the accumulated protein load becomes diluted by cell division. This scenario in neurons, which are differentiated, non-proliferating and high energetic demanding cells, might turn to be critical, compromising the turnover of proteins and organelles for proper cell functioning. Hence, several studies point to the fact that the overexpression of aggregation-prone proteins readily overwhelms the ability of the neuron to effectively clear aggregates by autophagy, e.g., [36,40,48]. Moreover, a direct function of SUMOylation in promoting the solubility of aggregation-prone proteins has been described [49,50]. Whether suboptimal protein degradation may contribute to disease pathogenesis in C9ALS/FTD is an appealing area that remains to be thoroughly investigated. As our knowledge about the mechanisms behind inclusion formation in neurodegenerative disorders improves, more specific and efficient treatments can be developed.

## Figures and Tables

**Table 1:** Clinical and molecular characterization of the patients and controls included in the study.

	Sex	Age (Years)	G4C2	Clinical Diagnosis
C9ORF72_1	Female	70	2, >145	FTD
C9ORF72_2	Male	76	2, >145	ALS/FTD
C9ORF72_3	Female	65	2, >145	ALS/FTD
C9ORF72_4	Female	69	2, >145	FTD
Control_1	Female	82	2, 2	Control
Control_2	Male	57	2, 6	Control
Control_3	Male	86	2, 4	Control
Control_4	Female	68	2, 2	Control

**Table 2:** Enzymatic activities of the mitochondrial respiratory chain in the fibroblasts from C9ALS/FTD patients.

**Enzyme** **Activities ^1^**	**Fibroblasts**				
	**C9ORF72_1**	**C9ORF72_2**	**C9ORF72_3**	**C9ORF72_4**	**Control Range**
Complex I+III	17.4	9.6 *	0 *	25.6	15–42
Complex II	19.2 *	34	18.1 *	40.6	22–35
Complex II+III	3.4 *	9.8 *	1.8 *	15.8	11–20
Complex III	4.6 *	8.3 *	0 *	14.2 *	25–48
Complex IV	67	42.2	18.1 *	68.5	33–57
Citrate synthetase (CS)	18.7 *	54	15.8 *	61.8	41–62
**Enzyme** **Activities ^2^**	**C9ORF72_1**	**C9ORF72_2**	**C9ORF72_3**	**C9ORF72_4**	**Control Range**
Complex I+III/CS	0.930	0.178 *	0 *	0.414	0.24–0.85
Complex II/CS	1.027	0.630	1.146	0.657	0.53–0.75
Complex II+III/CS	0.182 *	0.181 *	0.114 *	0.256	0.25–0.42
Complex III/CS	0.246 *	0.154 *	0 *	0.230 *	0.49–1.06
Complex IV/CS	3.583	0.781	1.146	1.108	0.84–1.15

^1^ nmol min^−1^ mg^−1^ protein; ^2^ nmol min^−1^ mg^−1^ protein enzyme activity expressed in percentage respect to CS activity; * Values below the control range.

**Table 3:** Autophagy-related and SUMOylation-related Reactome pathways deregulated in C9ALS/DTF patients compared to the controls.

PATH_ID	PATH_NAME	Size	padj	LOR
R-HSA-1632852	Macroautophagy	120	0.00004267	−0.4304
R-HSA-9612973	Autophagy	132	0.0002208	−0.3759
R-HSA-9663891	Selective autophagy	67	0.0005462	−0.4965
R-HSA-3108232	SUMO E3 ligases SUMOylate target proteins	151	0.000003633	0.4239
R-HSA-2990846	SUMOylation	157	0.000006105	0.4079
R-HSA-983168	Antigen processing: Ubiquitination and Proteasome degradation	284	0.004528	−0.2058
R-HSA-8852135	Protein ubiquitination	63	0.005484	−0.4211

LOR: logarithm of the odds ratio.

## Data Availability

All of the data is contained within the article.
